# Occurrence of *Fusarium* spp. and Fumonisins in Stored Wheat Grains Marketed in Iran

**DOI:** 10.3390/toxins2122816

**Published:** 2010-12-13

**Authors:** Khosrow Chehri, Saeed Tamadoni Jahromi, Kasa R. N. Reddy, Saeed Abbasi, Baharuddin Salleh

**Affiliations:** 1 Department of Plant Pathology, College of Agriculture, Razi University, Kermanshah, Iran; Email: abbasikhs@yahoo.com; 2 Persian Gulf and Oman Sea Ecological Research Institute, Bandar Abbas, Iran; Email: hadadi30@yahoo.com; 3 School of Biological Sciences, Universiti Sains Malaysia, 11800 USM, Penang, Malaysia; Email: sallehb@usm.my

**Keywords:** wheat, *Fusarium* spp., fumonisins, Iran

## Abstract

Wheat grains are well known to be invaded by *Fusarium* spp. under field and storage conditions and contaminated with fumonisins. Therefore, determining *Fusarium* spp. and fumonisins in wheat grains is of prime importance to develop suitable management strategies and to minimize risk. Eighty-two stored wheat samples produced in Iran were collected from various supermarkets and tested for the presence of *Fusarium* spp. by agar plate assay and fumonisins by HPLC. A total of 386 *Fusarium* strains were isolated and identified through morphological characteristics. All these strains belonged to *F. culmorum*, *F. graminearum*, *F. proliferatum* and *F.* *verticillioides*. Of the *Fusarium* species, *F. graminearum* was the most prevalent species, followed by *F. verticillioides*, *F. proliferatum* and then *F. culmorum*. Natural occurrence of fumonisin B1 (FB1) could be detected in 56 (68.2%) samples ranging from 15–155 μg/kg, fumonisin B2 (FB2) in 35 (42.6%) samples ranging from 12–86 μg/kg and fumonisin B3 (FB3) in 26 (31.7%) samples ranging from 13–64 μg/kg. The highest FB1 levels were detected in samples from Eilam (up to 155 μg/kg) and FB2 and FB3 in samples from Gilan Gharb (up to 86 μg/kg and 64 μg/kg).

## 1. Introduction

Wheat is one of the most important cereal crops for human consumption [1]. *Fusarium* species are responsible for many economically important cereal diseases such as head blights, ear rots and stem diseases, resulting in severe reductions in crop yield by up to 40% [2]. *F. graminearum*, *F. culmorum*, *F. avenaceum*, *F. chlamydosporum*, and *F. verticillioides*, which are the most important *Fusarium* species in central Europe and in large areas in North America and Asia, reduced both crop yield and cereal quality. In addition, *Fusarium* spp. are well known to produce fumonisins on cereal grains when environmental conditions are favorable for their growth under field and storage conditions [3,4,5]. Fumonisins are a group of structurally related toxic compounds produced mainly by *F. verticillioides* as well as by *F. proliferatum*, *F. napiforme* and *F. nygamai* [6,7]. 

Fumonisins have been reported to cause diseases in humans and animals after consumption of contaminated food and feed [8]. Diseases include equine leukoencephalomalacia, porcine pulmonary edema, hydrothorax in pigs, and gastric hemorrhages and hepatitis in rats [9]. Fumonisin B1 (FB1) has been implicated in human esophageal cancer; Sydenham *et al.* [10] reported FB1 concentrations were statistically correlated with esophageal cancer rates in Transkei, South Africa. There is an abundance of information concerning *Fusarium* species associated with wheat in the field [2,4,11,12,13,14], but there are few reports concerning *Fusarium* species associated with wheat in stored grains [14,15]. 

The occurrence of *F. verticillioides* and other toxigenic *Fusarium* species and the production of fumonisins in crops and their products are partly determined by environmental factors in the field, and during transportation and storage. Recently, Ghiasian *et al.* [16] reported the natural occurrence of *F. verticillioides* and fumonisins in corn from Iran. In another study, Dastmalchi [17] reported no detectable levels of aflatoxins in wheat from Iran; several other reports are available on aflatoxins in pistachio nuts [18,19] and aflatoxin M_1_ in milk [20,21,22] in Iran. However, no attempts have been made to determine the *Fusarium* spp. and fumonisins in wheat grains used for human consumption in Iran. The objectives of this study were (1) to isolate and identify *Fusarium* spp., and (2) to determine natural occurrence of fumonisins in wheat grains collected from various places in Iran.

## 2. Experimental

### 2.1. Collection of Wheat Samples and Mycological Analyses

Eighty-two stored wheat samples produced in Iran were collected from supermarkets (Table 1). *Fusarium* counts were determined by dilution plating and direct plating according to Ghiasian *et al.* [16] with minor modifications. Briefly, ground samples (10 g each) were mixed with 90 mL of sterile solution containing 0.1% peptone for the 10^−1^ dilution. Further serial dilutions to the 10^−5^ were made with 0.1% peptone solution. Aliquots (1.0 mL) of each dilution were then transferred to Petri dishes containing potato dextrose agar (PDA) under sterile conditions. The Petri dishes were incubated at 25 °C for 7 days. Colonies that developed on plates were counted at end of the incubation period and recorded as Colony Forming Units per gram (CFU/g). *Fusarium* incidence was also evaluated by direct plating. Briefly, kernels from each sample were surface disinfected for 1 min with 1% sodium hypochlorite solution, rinsed twice in sterile distilled water, and dried under sterile conditions. One hundred kernels per sample were plated onto pentachloronitrobenzene (PCNB) agar plates [23]. The plates were incubated at 28 °C for 5–7 days. The resulting *Fusarium* colonies recovered in both methods were identified based on morphological characters according to Nelson *et al.* [24] and Leslie and Summerell [25].

### 2.2. Extraction and Clean up of Fumonisins from Wheat Samples

Fumonisins were extracted and estimated according to the method of Sydenham *et al.* [10]. Briefly, 20 g of homogenized samples were mixed with 100 mL of 70% methanol and blended for 3 minutes. After centrifugation at 1,500 g for 10 min at 4 °C, the mixture was filtered through a Macherey-Nagel 617 filter paper. The pH was adjusted to 6.0 with 1 M NaOH and an aliquot (5 mL) was applied at a flow rate of less than 2 mL/min to a strong anion exchange (SAX) solid phase extraction cartridge (Bond-Elut, Varian, Harbor City, CA, USA) containing 500 mg sorbent. The cartridge was conditioned by successive passage of methanol (5 mL) and methanol/water (3:1, v/v, 5 mL). The columns were pre-washed with 5 mL of 100% methanol followed by 5 mL of methanol/water (75:25). Fumonisins were eluted from the columns with 1% acetic acid in 10 mL of methanol, dried in an evaporator, and used for HPLC analysis. 

### 2.3. Determination of Fumonisins by HPLC Analysis

Fumonisin standards were obtained from Sigma, St Louis, USA. Standard solutions were prepared containing FB1 (245 µg/mL), FB2 (200 µg/mL) and FB3 (285 µg/mL) in acetonitrile/water (1:1, v/v) [16] and determined by HPLC analysis using fluorescence detection was conducted as described by Sydenham *et al.* [10]. An aliquot (25 µL) of purified extract was derivatized with 225 µL *O*-phthaldialdehyde solution prepared by dissolving 40 mg of *O*-phthaldialdehyde in 1 mL of 100% methanol, diluting into 25 mL of distilled water containing 5 mL of 0.1 M disodium tetraborate, and mixing with 50 µL of 2-mercaptoethanol. The fumonisin *O*-phthaldialdehyde derivatives were analyzed using HPLC system consisting of a Waters model 590 pump (Milford, MA) connected to a Waters 470 scanning fluorescence detector. The separations were performed on a reversed phase stainless steel Ultracarb 5 ODS column (150 mm × 4.6 mm id, Phenomenex, Torrance, CA). The mobile phase was methanol/0.1 M sodium dihydrogen phosphate (75:25, v/v) adjusted to pH 3.35 with ortho-phosphoric acid and pumped at a flow rate of 1 mL/min. Fumonisins were quantified by peak area measurement in comparison with fumonisin reference standard using Borwin integration software (JMBS Developments, LE Fontanil, France). Recoveries were determined in uncontaminated wheat samples spiked at levels of 100, 500 and 1000 μg/kg. Mean recoveries with SDs were 82.1 ± 2.02, 83.5 ± 1.95 and 86.9 ± 3.12 for FB1, FB2 and FB3, respectively. The detection limit was 10 μg/kg.

## 3. Results and Discussion

### 3.1. Fusarium spp. in Wheat Grains

Eighty-two wheat samples produced in Iran were analyzed for the occurrence of *Fusarium* spp. and fumonisins. All samples were found positive for *Fusarium* species. A total of 386 strains were isolated and identified using morphological characters. All these strains belonged to four *Fusarium* spp.; *F. graminearum, F. culmorum, F. proliferatum* and *F*. *verticillioides* (Table 1). *F. graminearum* was the most prevalent species in all samples followed by *F. verticillioides*, *F. proliferatum* and *F. culmorum*. *F. culmorum* was not observed in the samples collected from Eilam, Gilan Gharb, Qasr Shirn and Sarpol Zohab (Table 1).

**Table 1:** Occurrence of *Fusarium* spp. in wheat grains from Iran.

Place of Sample Collection	Samples Analyzed	*Fusarium* spp. (cfu/g × 10^3^)^a^				Kernels Infected (%)^a^			
		*F. gr*	*F. ve*	*F. cu*	*F. pr*	*F. gr*	*F. ve*	*F. cu*	*F. pr*
Asad Abad	3	33.2	26.3	10.5	12.3	35	33	14	18
Bisotun	8	35.4	27.2	12.2	14.2	40	35	9	16
Eilam	2	27	18	0	12	33	22	0	14.2
Gilan Gharb	9	40.2	28.3	0	15.2	53.3	48.2	0	26.2
Gorveh	6	24.2	20.2	12.2	12	28.2	24.2	14.2	12.3
Hamadan	8	33.6	28	10.2	11.2	41.2	38	12	5
Kamyaran	4	34.4	22.2	12.4	12.2	33.3	33	8	20
Kermanshah	6	25.4	24.2	12.2	16.2	43.2	45	12	18
Kangavar	3	29.2	26.2	10.2	0	34.3	25	16.4	0
Kurdistan	7	29	30.2	11.2	14.2	38.3	34	16.2	13
Qasr Shirn	4	37	16	0	15.5	43	37	0	23.3
Ravansar	8	40	36.2	10.5	17	54	43	12.2	15
Sarpol Zohab	3	35	37	0	16	52	44	0	26
Sahneh	6	28.4	34	10.2	14	45	38	16.4	18
Sonqor	5	22	33	12.2	0	26.2	25.6	19.6	0

^a^ average of all positive samples; Abbreviations: *F. gr = F. graminearum; F. ve = Fusarium verticillioides; F. cu = Fusarium culmorum; F. pr = Fusarium proliferatum.*

*F. graminearum* is one of the most frequently found *Fusarium* species on European cereals, where it is more common in the wet and warm climate of central and Southern Europe [26]. A species commonly found in wheat grown in cooler regions is *F. culmorum* [27,28,29,30,31,32]. In this study, we also identified *F. graminearum* isolates in all samples from the warm regions, mainly Kermanshah, Hamadan, Kurdistan and Eilam provinces, and *F. culmorum* isolates were only found in samples from moderate to cold regions of Iran (Table 1). 

The occurrence of mycotoxins produced by *Fusarium* spp. in small cereal grains, particularly in wheat, is of great concern worldwide, because their presence in processed feeds and foods seems unavoidable. Consequently, they have been associated with chronic or acute mycotoxicoses in livestock and, to a lesser extent, in humans [2,33]. In this survey, the members of *Gibberella* *fujikuroi* complex, especially *F. verticillioides* and *F. proliferatum,* were detected at the highest frequencies after *F. graminearum* (Table 1). Our results are in agreement with other studies in the USA, Canada, Argentina [34,35] and Europe [2,11]. 

### 3.2. Fumonisins in Wheat Grains

Of the 82 samples analyzed, 56 (68.2%) were found positive for FB1 contamination ranging from 15–155 μg/kg. The greatest FB1 contamination was detected in samples collected from Eilam ranging from 22–155 μg/kg followed by samples from Kangavar ranging from 65–142 μg/kg (Table 2). All samples (100%) from Asad Abad and Gilan Gharb were contaminated with FB1 ranging from 25–85 μg/kg. FB2 was detected in 35 (42.6%) samples ranging from 12–86 μg/kg. FB2 was not detected in samples collected from Kangavar (Table 2). FB3 could be detected in 26 (31.7%) samples ranging from 13–64 μg/kg. Highest FB2 and FB3 were detected in samples collected from Gilan Gharb ranging from 38–86 μg/kg and 25–64 μg/kg, respectively. FB3 was not detected in samples collected from Eilam, Kangavar, Sarpol Zohab and Sonqor. 

**Table 2:** Fumonisins detected in wheat grains from Iran.

Place of Sample Collection	Samples Analyzed	FB1	FB2	FB3	Total Fumonisins
		Positive Samples/Range (μg/kg)	Positive Samples/Range (μg/kg)	Positive Samples/Range (μg/kg)	Range( μg/kg)
Asad Abad	3	3/25–62	3/16–28	2/16–24	57–114
Bisotun	8	5/19–71	4/14–25	4/21–28	54–124
Eilam	2	2/22–155	1/56	nd	78–155
Gilan Gharb	9	9/28–85	7/38–86	7/25–64	91–235
Gorveh	6	4/85–113	2/25–34	2/13–26	123–173
Hamadan	8	5/19–41	2/12–26	2/18–21	49–88
Kamyaran	4	3/15–34	3/25–36	2/13–32	53–102
Kermanshah	6	4/85–123	2/15–28	1/18	118–151
Kangavar	3	2/65–142	nd	nd	65–142
Kurdistan	7	4/15–34	3/14–25	3/14–23	43–82
Qasr Shirn	4	2/25–85	1/15	1/16	56–85
Ravansar	8	6/21–32	3/14–29	3/15–22	50–83
Sarpol Zohab	3	2/25–83	1/35	nd	60–83
Sahneh	6	4/23–36	4/16–35	2/15–29	54–100
Sonqor	5	2/18–23	1/12–46	nd	30–69
Total	82	56/15–155	35/12–86	26/13–64	30–235

In a survey of ethnic foods in the United Kingdom, one of four wheat based noodles was reported to contain 26 µg/kg fumonisins [36]. A survey of fumonisin contamination in cereals conducted in Spain reported FB1 in 8 of 17 wheat samples in the range of 0.2–8.8 mg/kg and FB2 in one sample (0.2 mg/kg) [37]. Our results show that 68.2% of wheat samples were contaminated with FB1 ranging from 15 to 155 ug/kg, 42.6% of samples were contaminated with FB2 ranging from 12 to 86 μg/kg, and 31.7% samples for FB3 ranging from 13–64 μg/kg. Of the 42 white wheat flour samples analyzed in Italy, five were contaminated at levels below 100 µg/kg; of the 214 white wheat flour samples from France, 76 were reported to be contaminated, mostly at levels below 100 µg/kg [26]. However, until today, no attempts have been made to elucidate the fumonisin levels in wheat grains and its products in Iran. 

## 4. Conclusion

The results of this study showed that wheat grain samples collected from different supermarkets in Iran varied in their fungal distribution and fumonisin contamination. The overall results demonstrate that 68.2%, 42.6% and 31.7% of wheat samples showed contamination by FB1, FB2 and FB3 ranging from 15–155 μg/kg, 12–86 μg/kg and 13–64 μg/kg, respectively. The fumonisin levels detected in all wheat samples were below the permissible limits (<1,000 μg/kg) for human consumption. However, further studies are warranted to analyze all mycotoxins which have impact on human and animal health in a large number of wheat products available in Iran to develop proper management strategies. 

## References

[1] Farshadfar E., Ghasempour H., Vaezi H. (2008). Molecular aspects of drought tolerance in bread wheat (*T. aestivum*). Pakistan J. Biol. Sci..

[2] Bottalico A., Perrone G. (2002). Toxigenic *Fusarium* species and mycotoxins associated with head blight in small grains cereals in Europe. Eur. J. Plant Pathol..

[3] McMullen M.P., Jones R., Gallenberg D. (1997). Scab of wheat and barley: A re-emerging disease of devastating impact. Plant Dis..

[4] Yli-Mattila T. (2010). Ecology and evolution of toxigenic *Fusarium* species in cereals in northern Europe and Asia. J. Plant Pathol..

[5] Gamanya R., Sibanda L. (2001). Survey of *Fusarium moniliforme* (*F. verticillioides*) and production of fumonisin B in cereal grains and oilseeds in Zimbabwe. Int. J. Food Microbiol..

[6] Nelson P.E., Plattner R.D., Shackelford D.D., Desjardins A.E. (1991). Production of fumonisins by strains of *Fusarium moniliforme* from various substrates and geographic areas. Appl. Environ. Microbiol..

[7] Thiel P.G., Marasas W.F.O., Sydenham E.W., Sheperd G.S., Gelderblom W.C.A., Niewenhuis J.J. (1991). Survey of fumonisin production by *Fusarium* species. Appl. Environ. Microbiol..

[8] Reddy K.R.N., Salleh B., Saad B., Abbas H.K., Abel C.A., Shier W.T. (2010). An overview of mycotoxin contamination in foods and its implications for human health. Toxin Rev..

[9] Bucci T.J., Howard P.C. (1996). Effect of fumonisin mycotoxins in animals. Toxin Rev..

[10] Sydenham E.W., Shephard G.S., Thiel P.G. (1992). Liquid chromatographic determination of fumonisin B1, B2 and B3 in foods and feeds. J. AOAC Int..

[11] Torp M., Nirenberg H.I. (2004). *Fusarium langsethiae* sp. on cereals in Europe. Int. J. Food Microbiol..

[12] Darvishnia M., Alizadeh A., Mohammadi Goltapeh E., Zare R. (2006). Three new *Fusarium* taxa isolated from gramineous plants in Iran. Rostaniha.

[13] Davari M., Didar-Taleshmkaeil R., Hajieghrari B. (2006). Wheat *Fusarium* head blight and identification of dominant species in Moghan area, Iran. Comm. Agric. Appl. Biol. Sci..

[14] Kachuei R., Yadegari M.H., Rezaie S., Allameh A.A., Safaie N., Zaini F., Khanezad Y.F. (2009). Investigation of stored wheat mycoflora, reporting the *Fusarium* cf. *langsethiae* in three provinces of Iran during 2007. Ann. Microbiol..

[15] Magan N., Aldred D., Mylona K., Lambert R.J.W. (2010). Limiting mycotoxins in stored wheat. Food Addit. Contam..

[16] Ghiasian S.M., Maghsood A.H., Yazdanpanah H., Shephard G.S., van der Westhuizen L., Vismer H.F., Rheeder J.P., Marasas W.F.O. (2006). Incidence of *Fusarium verticillioides* and fumonisins in corn from main production areas in Iran. J. Agric. Food Chem..

[17] Dastmalchi A. (2008). Survey and determination of aflatoxins in Iranian wheat of canvas covered store and variations of them after storage. Res. J. Biol. Sci..

[18] Cheraghali A.M., Yazdanpanah H., Doraki N., Abouhossain G., Hassibi M., Aliabadi S., Alikbarpoor M., Amirahmadi M., Askarian A., Fallah N., Hashemi T., Jalali M., Kalantari N., Khodadadi E., Maddah B., Mohit R., Mohseny M., Phaghihy Z., Rahmani A., Setoodeh L., Soleimany E., Zamanian F. (2007). Incidence of aflatoxins in Iran pistachio nuts. Food Chem. Toxicol..

[19] Pour R.S., Rasti M., Zighamian H., Garmakhani A.D. (2010). Occurrence of aflatoxins in pistachio nuts in Esfahan Province of Iran. J. Food Safe..

[20] Alborzi S., Pourabbas B., Rashidi M., Astaneh B. (2006). Aflatoxin M_1_ contamination in pasteurized milk in Shiraz (south of Iran). Food Control.

[21] Tajkarimi M., Aliabadi-Sh F., Nejad A.S., Poursoltani H., Motallebi A.A., Mahdavi H. (2008). Aflatoxin M_1_ contamination in winter and summer milk in 14 states in Iran. Food Control.

[22] Heshmati A., Milani J.M. (2008). Contamination of UHT milk by aflatoxin M_1_ in Iran. Food Control.

[23] Nash S.M., Snyder W.C. (1962). Quantitative and estimations by plat counts of propagules of the bean rot *Fusarium* in field soils. Phytopathology.

[24] Nelson P.E., Toussoun T.A., Marasas W.F.O. (1983). Fusarium Species: An Illustrated Manual for Identification.

[25] Leslie J.F., Summerell B.A. (2006). The Fusarium Laboratory Manual.

[26] Nicholson P., Chandler E., Draeger R.C., Gosman N.E., Simpson D.R., Thomsett M., Wilson A.H. (2003). Molecular tools to study epidemiology and toxicology of *Fusarium* head blight of cereals. Eur. J. Plant Pathol..

[27] Parry D.W., Jenkinson P., Mcleoad L. (1995). Fusarium ear blight (scab) in small grain cereals. A review. Plant Pathol..

[28] Snijders C.H.A. (1990). Fusarium head blight and mycotoxin contamination of wheat. Plant Pathol..

[29] Dawson W.A.J.M., Jestoi M., Rizzo A., Nicholson P., Bateman G.L.  (2004). Field evaluation of fungal competitors of *Fusarium culmorum* and *F. graminearum*, causal agents of ear blight of winter wheat, for control of mycotoxin production in grain. Biocontr. Sci. Technol..

[30] (2003). Collection of occurrence data of Fusarium toxins in food and assessment of dietary intake by the population of EU member states; Reports on tasks for scientific cooperation.

[31] Wong L.S.L., Tekauz A., Leisle D., Abramson D., Mckenzie R.I.H. (1992). Prevalence, distribution and importance of *Fusarium* head blight in wheat in Manitoba. Can. J. Plant Pathol..

[32] Wilcoxon R.D., Kommedall T., Ozmon E.A., Windels   C. (1988). Occurrence of *Fusarium* species in scaby wheat from Minnesota and their pathogenicity to wheat. Phytopathology.

[33] Desjardins A.E. (1995). Population structure of *Gibberella pulicaris* (anamorph, *Fusariums ambucinum*) from potato tuber dry rot in North America and Europe. Am. Potato J..

[34] Leslie J.F., Pearson C.A.S., Nelson P.E., Toussoun T.A. (1990). *Fusarium* species from corn, sorghum and soybean fields in the central and eastern United States. Phytopathology.

[35] Burgess L.W., Summerell B.A. (1992). Mycogeography of *Fusarium* species in subtropical and semi-arid grassland soils from Queensland. Aus. Mycol Res..

[36] Yli-Mattila T., Paavanen-Huhtala S., Parikka P., Konstantinova P., Gagkaeva T.Y. (2004). Molecular and morphological diversity of *Fusarium* species in Finland and northwestern Russia. Eur. J. Plant Pathol..

[37] IARC (1993). Some Naturally Occurring Substances: Food Items and Constituents, Heterocyclic Aromatic Amines and Mycotoxins. IARC Monographs on the Evaluation of Carcinogenic risks to Humans.

[38] Walker S.L., Leath S., Hagler W.M., Murphy J. (2001). Variation among isolates of *Fusarium graminearum* associated with Fusarium head blight in North Carolina. Plant Dis..

[39] González H.H.L., Molto G.A., Pacin A., Resnik S.L., Zelaya M.J., Masana M., Martínez E.J. (2008). Trichothecenes and mycoflora in wheat harvested in nine locations in Buenos Aires Province, Argentina. Mycopathologia.

[40] Patel S., Hazel C.M., Winterton A.G.M., Mortby E. (1996). Survey of ethnic foods for mycotoxins. Food Addit. Contam..

[41] Castella G., Bragulat M.R., Cabanes F.J. (1999). Surveillance of fumonisins in maize-based feeds and cereals from Spain. J. Agric. Food Chem..

